# Direct fluorescence antibody testing augments syphilis diagnosis, compared to serology alone

**DOI:** 10.1177/09564624211048610

**Published:** 2021-11-01

**Authors:** Lauren Orser, Patrick O’Byrne

**Affiliations:** 1 University of Ottawa, 6363 School of Nursing, Ottawa, ON, Canada; 2 Ottawa Public Health, 6363 Sexual Health Clinic, Ottawa, ON, Canada

**Keywords:** syphilis, testing, sexually transmitted infections

## Abstract

In Ottawa, Canada, we initiated protocols to include non-serologic syphilis testing, as direct fluorescence antibody (DFA) for patients with syphilis symptoms. The purpose was to assess the ability of DFA to detect syphilis during acute infection and to determine if non-serologic testing could yield an increased number of syphilis diagnoses. We reviewed charts of patients of our local sexual health clinic for whom syphilis was suspected. A total of 69 clinical encounters were recorded for 67 unique patients, most of whom were male. The most common symptom was a painless genital lesion. Of the 67 patients, 29 were found to have a new syphilis diagnosis, among whom, 52% had positive syphilis serology and positive DFA, 34% had a positive syphilis serology and negative DFA, and 14% had negative syphilis serology and positive DFA. While DFA testing did not yield an abundance of new cases, it was useful to support findings from syphilis serology or confirm diagnosis where serology was negative. Where available, alternate non-serologic tests, such as nucleic acid amplification tests, should be considered above DFA due to its higher sensitivity for detecting syphilis in primary lesions; however, in clinical situations, when new syphilis infection is suspected, empiric treatment should not be delayed.

## Introduction

The overall rate of syphilis in Ontario was 5.9/100,000 in 2010 and increased to 16.3/100,000 by 2019.^1^ For men, this change was from 11.3/100,000 in 2010 to 30.7/100,000 by 2019; for women, this rate rose from 0.7/100,000 in 2010 to 2.1/100,000 in 2019.^1^ These increased rates occurred in concordance with more people presenting for care with potential symptoms of primary syphilis. While a simple solution could be to promote empiric treatment for syphilis at the point of care, the issue is that serology might not detect a syphilis infection for upwards of four to 6 weeks after exposure.^2,3^ This means that in some cases, there is no conclusive diagnosis of primary syphilis, resulting in a lack of contact tracing of sexual partners or appropriate clinical follow-up for these persons, per extant clinical guidelines.^4^ This situation also affects surveillance data about syphilis, potentially resulting in underreporting of infectious syphilis cases.

To address this, we began collecting specimens for direct fluorescence antibody (DFA) testing from patients who presented to our sexual health clinic with symptoms suspicious for syphilis and tracked the results. Our aims were (1) to assess the utility of DFA testing to confirm clinical suspicions of syphilis and (2) to determine if, and at what rate, DFA would detect positive syphilis results in the absence of confirmatory serology. We hoped DFA testing would improve our diagnostic capabilities to better identify primary syphilis to facilitate rapid treatment for patients and their sexual contacts and to strengthen local public health surveillance of infectious syphilis cases. As part of this review, we manually tracked DFA tests completed in our clinic and compared the findings against syphilis serology results to determine whether the use of DFA would increase the number of laboratory-confirmed syphilis diagnoses. The results of this review are reported here.

### Background

Syphilis is a sexually transmitted infection caused by the bacterium, *Treponema Pallidum (T. Pallidum)*.^5^ In its primary phase, syphilis invades local, healthy tissue causing inflammation and consequential erosion of the genital, perineal, rectal, or oral mucosa.^5,6^ During this phase, lesions known as chancres appear at the site of infection, whether genital, oral, or anal. Chancres classically present as single, round, ulcerative lesions with indurated margins and a non-exudative base, although multiple or painful lesions have been reported, though usually in patients with chronic immune conditions.^7^ Regional lymphadenopathy can also occur. Within approximately 90 days, syphilis then progresses to its secondary stage, which denotes systemic dissemination.^5^ This phase of infection can last up to 6 months. Most commonly, patients present with an erythematous to copper-colored maculopapular rash to the palms of hands, soles of feet, and/or trunk, as well as non-specific symptoms, patchy alopecia, and cranial nerve dysfunction (specifically II, VI, VII, and VIII).^5–7^ Within a period of a year, syphilis progresses to its latent phase. In the early latent stage, patients are generally asymptomatic and only diagnosed with blood testing; however, relapses to the secondary phase occur in about 25% of early latent cases.^6^ Following this 1-year period, syphilis transitions to a late latent phase, where the infection is considered non-infectious and thus non-transmissible to sexual partners. Left untreated, syphilis can cause damage to the cardiovascular, neurologic, and integumentary systems, which in some cases can lead to irreversible health complications, such as vision or hearing loss, meningitis, or damage to the aorta.^5–7^ For this reason, early identification and treatment of syphilis is prudent to reduce negative sequalae of infection.

In Ontario, where this review occurred, syphilis is primarily diagnosed by serologic testing using the reverse screening algorithm.^8^ For this, samples are screened using chemiluminescent microparticle immunoassay (CMIA), an automated test for treponemal detection of IgG and IgM antibodies.^8,9^ If the CMIA is non-reactive, no further testing occurs. If the CMIA is reactive, specimens are automatically tested using a quantitative rapid plasma reagin (RPR). At the time of this review, specimens with a non-reactive RPR are subject to a second treponemal test using the Treponema pallidum Particle Agglutination (TPPA)^8^ to confirm syphilis diagnosis.

To stage syphilis and to guide management decisions, clinicians must not only rely on syphilis serology results but also assess for symptoms, risk behavior, and changes in RPR titers to determine if an infection is recent (i.e., primary, secondary, and early latent) or older (i.e., late latent and tertiary). One issue with the reverse screening algorithm is that the RPR can take 4–6 weeks to change following exposure.^4^ As a result, patients with a history of syphilis who present with symptoms of primary infection may not receive appropriate management if diagnosis is made based solely on serology. Further challenging syphilis diagnosis is that chancres are often painless and present in locations (e.g., rectum, oral cavity, and labia), where they are not easily detected.^7^ Moreover, when symptoms are visible, it can be difficult to differentiate a chancre from other dermatologic eruptions (e.g., herpes simplex, chancroid, or lymphogranuloma venereum, among others).^10^

In addition to serology, some laboratories offer non-serologic syphilis tests, designed to detect *T. Pallidum* from lesions. These tests include (1) nucleic acid amplification testing (NAAT), which has a sensitivity of 93.1–100% in primary syphilis and specificity of 100%^11–13^ and can be used on genital, oral, and rectal lesions; (2) dark-field microscopy, which has a sensitivity of 86–97% and specificity of 100%^9^ and examines the morphology and movement of live spirochetes in genital lesions; and (3) DFA, which has a sensitivity of 73–100% and specificity of 100%^9^ and can be used on lesions of all sites, but is limited by potential cross-reactivity with non-pathogenic treponemal organisms in the oral and rectal mucosa.^4,9^ While NAAT is considered to be a superior non-serologic testing approach due to its increased sensitivity to detect primary syphilis,^12–14^ in Ontario, where this review occurred, this test is not available.^8^ For this reason, DFA was used for this study, despite reduced sensitivity on exudate from primary or secondary genital lesions^9,11^ and limited validation and approval for use outside of select Canadian provinces.^13^

## Methods

This review occurred in Ottawa, Ontario, among patients who presented to our sexual health clinic from 28 June 2018 to 31 December 2020. Pre-COVID, this clinic would see approximately 20,000 patients per year and would account for nearly one-third of all local syphilis diagnoses.^15,16^ As part of this review, we established protocols for physicians and nurse practitioners to complete DFA tests on patients where a diagnosis of syphilis was suspected. Protocols included indications for testing (e.g., symptoms of concern, anatomical site of lesions) and instructions on how to complete a DFA, per Public Health Ontario Laboratory recommendations.^8^ Informational sessions were held to provide hands-on guidance of clinical procedures. All completed DFA tests were logged on a tracking sheet, which included patients’ information and indication for testing.

In Ontario, a diagnosis of syphilis is reportable—meaning that all results with a reactive CMIA are automatically reported to local public health departments, regardless of the reported non-treponemal level (i.e., RPR quantitation).^17,18^ For new syphilis diagnoses, whether infectious or non-infectious, health units complete follow-up with patients to ensure they have received appropriate treatment as indicated in STI guidelines and contact tracing to ensure that sexual partners of positive cases receive testing and treatment for syphilis.^18^ Counselling regarding syphilis transmission, treatment, and management, as well as indications for repeat syphilis testing are also provided as part of public health follow-up for patients diagnosed with syphilis.

We manually reviewed the health records of patients who presented to the sexual health clinic from 28 June 2018 to 31 December 2020 for whom a DFA was completed. In addition, we collected information on patient demographics (age, gender, and sexual orientation), symptomatology at point of care, and final test results. For DFA tests, results were logged as positive or negative based on presence or absence of *T. Pallidum*. For syphilis serology, we reported presence of CMIA, RPR measurement, and TPPA (if applicable). For patients who had a prior history of syphilis, we compared clinical findings against RPR results to determine if results were new (logged as primary infection) or consistent with a prior infection (logged as “previous positive”).^19^ Because this review exclusively reports on data collected during mandated public health follow-up, research ethics approval was not required. In addition, all patients who present to the sexual health clinic provide written consent for the use of health information as part of clinical reviews. This study was thus considered to be a negligible risk of harm to participants.

### Findings

During the study period, we had 69 unique testing episodes from a total of 67 patients. Almost all patients in this review identified as male (95.5% or *n* = 64/67) and were an average of 36 years old (range: 18–68 years). For risk factors, most male patients reported sex with same sex partners, while those who did not identify as male reported sex with opposite sex partners. Nine of the 67 patients in this review were reported to be HIV sero-positive; 33.3% (*n* = 3/9) of whom were simultaneously diagnosed with HIV and syphilis during the review period.

Of these 69 instances of testing, 29 had a positive syphilis serology test with a reactive CMIA screen. The remaining 40 cases either had negative syphilis serology and negative DFA or had a prior history of syphilis with a negative DFA. In both instances, since the DFA was negative, serology results showed no change RPR, and no patient had unequivocal symptoms of syphilis, cases were classified as “negative” or “previous positive.” Focusing on the 29 patients with positive syphilis laboratory results, 52% (*n* = 15) had a positive DFA result with a positive syphilis serology, 34% (*n* = 10) had a negative DFA result with positive serology, and 14% (*n* = 4) had positive DFA with negative syphilis serology. Of these 29 cases, 19 had positive DFA for a test sensitivity of 66% (95% CI: 49–83%). Serology, by comparison, was positive for 25 cases for a test sensitivity of 86% (95% CI: 74–98%). Of these 29 cases, 22 were diagnosed with primary syphilis and 7 with secondary syphilis. A full 100% (*n* = 4) of the cases when DFA was positive and serology was not were primary syphilis, while only 70% (*n* = 7) of the cases of positive serology and negative DFA were primary syphilis.

Among the 29 patients with a new syphilis infection, the most common symptom was genital lesion(s), which presented in 83% (*n* = 24/29) of cases. For patients with lesions, 11 were diagnosed as chancres at the point of care and 13 were classified as indurated lesions not yet diagnosed. The most common site of ulceration was the penis (*n* = 21/24), with few patients presenting with extragenital lesions. Notably, 16% of cases (*n* = 4/24) had multiple ulcerated lesions. The remaining five of 29 patients either presented with symptoms of primary and secondary syphilis concurrently (i.e., genital lesions and rash) or exclusively had secondary syphilis symptoms (e.g., rash, condyloma lata, and/or mucous patches on genitals or in the oral cavity).

## Discussion

The findings from this review raise some interesting points of consideration for clinical practice. The first relates to the number of new syphilis cases identified through positive DFA testing in the absence of positive syphilis serology, which accounted for 14% of newly diagnosed infections during the review period. While the number of cases is small, DFA testing was able to definitively diagnose an additional four patients who presented during a period of acute syphilis infection, when serology was unable to detect treponemal antibodies. DFA can, however, be less sensitive (particularly when applied to dry or healing lesions) and have mixed evidence for *T. Pallidum* detection in the oral and rectal mucosa.^9,11-13^ Considering this, clinicians must interpret a negative DFA result with caution and prioritize use of NAAT for syphilis diagnosis when available.^12,13^ In instances where patients’ risk factors and STI history are suspicious for primary infection at the point of care,^4,6,19^ prescribers should consider offering empiric treatment for syphilis knowing that such treatment may halt seroconversion of treponemal antibodies (IgG and IgM),^9^ which could yield negative serology results, but not mean the patient did not have syphilis. When syphilis is less likely, clinicians could alternatively consider completing syphilis serology at the visit and, if negative, repeating testing in 4 weeks to rule out infection.

The second point relates to the need for examination of patients who present with genital lesions. Because syphilis has protean manifestations which make it difficult to identify, its differential diagnosis is varied and includes herpes simplex virus, human papillomavirus, LGV, HIV seroconversion, chancroid, folliculitis, and balanitis, and other inflammatory skin lesions.^4,7,10^ As well, given the clinical variability of syphilis, clinicians should perform physical examination, rather than relying on history alone.^19^ For example, when a clinical diagnosis cannot be established at the point of care, prescribers should consider multiple tests to rule out a diagnosis, including syphilis serology and a DFA when primary syphilis is suspected, while noting that NAAT is a superior approach for detecting new syphilis infection, but is limited by lack of widespread availability in Canada and the US. Incorporating non-serologic testing (ideally using NAAT) into clinical examinations of symptomatic patients might not only lead to more conclusive diagnoses^12–14,20^ but also has the benefit of ensuring appropriate treatment and follow-up interventions. A positive non-serologic test as well, helps rule out other differential diagnoses (e.g., balanitis) and can facilitate diagnosis when positive in persons with a history of syphilis infection when the CMIA will remain reactive and the RPR may have not yet changed.

More robust syphilis diagnoses also have added public health benefits. The use of non-serologic syphilis tests, such as DFA or preferably NAAT, can help detect new syphilis diagnoses to trigger follow-up by public health departments, including rapid treatment of patients and their sexual contacts.^17,20^ In addition, cases diagnosed by non-serologic methods can provide a more accurate depiction of infectious syphilis within public health jurisdictions for the purpose of infection monitoring.^15^ This information can then be used to guide public health education and/or testing campaigns in areas or among sub-population groups where higher numbers of acute syphilis cases are clustered.^21–24^

Finally, our discussion of syphilis testing also warrants a review of follow-up considerations for patients who are diagnosed with syphilis. One recommendation is that all patients complete repeat syphilis testing at 3, 6, and 12 months following syphilis treatment.^4,6^ This is especially important considering that 5–10% of all syphilis treatments fail,^24^ meaning that even when patients receive first-line therapy for syphilis, it is possible their treatment will be ineffective. As part of follow-up, clinicians should assess symptoms at all testing visits and monitor RPR titers to ensure at least a four-fold decline in RPR titer within 6–12 months following treatment.^6^ These recommendations also apply to patients, such as the 4 cases in this review, who had positive DFA or other non-serologic tests with negative syphilis serology results. In this instance, clinicians should assess for symptoms of primary or secondary infection and repeat syphilis serology at the advised intervals to ensure these results remain negative; any deviation from the results at the point of treatment may be an indication of treatment failure or subsequent syphilis infection.

In addition to follow-up testing, clinicians should ensure patients with positive syphilis result receive screening for other sexually transmitted infections, including HIV, chlamydia, gonorrhea, and hepatitis B and C.^25^ This point is reinforced by the 4% (*n* = 3) of patients in this review who were concurrently diagnosed with syphilis and HIV after presenting to our clinic for assessment of genital symptoms. In all instances, patients were appropriately treated for syphilis and referred for ongoing HIV management and care. HIV sero-negative patients diagnosed with syphilis should receive counselling regarding HIV prevention, including counselling about and referrals for HIV pre-exposure prophylaxis (PrEP)^26^ due to the potential risk for HIV transmission caused by increased expression of target HIV cells in the genital mucosa during primary or secondary syphilis infection.^27,28^ The need for PrEP is further supported by estimates from the United States Centers for Disease Control and Prevention (CDC), which suggest that one in 18 men who have sex with men will acquire HIV within 12 months of being diagnosed with syphilis.^20^ For chlamydia and gonorrhea screening, clinicians should ensure that patients who are engaging oral and/or anal sex receive extragenital testing in addition to urine tests, given the high incidence of asymptomatic infections located in the pharynx and rectum.^29^

### Limitations

This review has a few limitations. First, it occurred in a single Canadian city and was exclusively run in a specialized clinic that sees a higher number of syphilis cases compared to other clinical care sites. Had this review occurred in multiple sites or a variety of clinical settings, the results might have differed. Nonetheless, this specialized focus potentially yields a better depiction of the utility of DFA testing. Second, while highly specific, DFA testing has poorer sensitivity, meaning that results could register as a false negative if insufficient exudate is collected from specimens. It is therefore possible that some of negative DFA results reported could have been positive. The training we provided to specialists in STI care, however, hopefully minimized the possible occurrence. Finally, COVID-19 restrictions arose during this review period, so it is possible patients with symptoms may have deferred care when lesions were no longer present, resulting in potentially missed or delayed diagnoses. Again, however, the results indicate the utility of adding DFA testing to serology for persons who present for care.

## Conclusions

In this review, we report on findings from an 18-month review of patients who presented to a sexual health clinic with symptoms suspicious of syphilis and completed serologic and non-serologic testing for syphilis, using a DFA. Results from this review found that the majority of new syphilis infections were identified by serology in combination with DFA; however, we did identify four new cases of syphilis using the DFA where serology reports were negative. Our findings suggest that while syphilis serology alone is likely to detect new syphilis infection, non-serologic testing can be useful to confirm suspicions of primary syphilis among patients with genital lesions. When available, however, NAAT should be used in lieu of DFA, given its superior sensitivity in detecting primary syphilis compared to DFA. Lastly, correct identification of diagnosis not only leads to improved treatment of the person with syphilis but also follow-up with partners and, as a whole, more comprehensive diagnosis of syphilis could yield improved individual and population health outcomes of an infection that is currently increasing in incidence worldwide.
